# Recognising dying in motor neurone disease: A scoping review

**DOI:** 10.1177/02692163241263231

**Published:** 2024-07-28

**Authors:** Elizabeth Abbey, Maimoona Ali, Matthew Cooper, Paul Taylor, Catriona R Mayland

**Affiliations:** 1 Division of Clinical Medicine, University of Sheffield, Sheffield, UK; 2 Sheffield Teaching Hospitals NHS Foundation Trust, Sheffield, UK; 3 The Medical School, University of Sheffield, Sheffield, UK; 4 School of Health and Related Research, University of Sheffield, Sheffield, UK; 5 St Luke’s Hospice, Sheffield, UK; 6 Palliative Care Unit, University of Liverpool, Liverpool, UK

**Keywords:** Palliative care, palliative supportive care, motor neuron disease, review

## Abstract

**Introduction:**

Timely identification of dying in motor neurone disease enables optimal care, yet we know that healthcare professionals can fail to recognise when death is approaching. Clinical factors help predict the end of life in other terminal conditions. Examining these principles in motor neurone disease would help guide more accurate recognition of this critical phase.

**Aim:**

To examine and map out what is known about dying in patients with motor neurone disease, and the recognition of dying by healthcare professionals.

**Design:**

A scoping review was conducted following the Arksey and O’Malley methodological framework.

**Data sources:**

Four electronic databases (MEDLINE, Scopus, PsycINFO and CINAHL) and grey literature were searched on the 10th May 2023. Reference lists and citations were also reviewed.

**Results:**

From 1512 articles, 13 studies were included. Dyspnoea, anxiety and pain were the most common symptoms associated with the dying phase. Worsening respiratory function, the development of specific new symptoms and deteriorating symptom control suggested approaching death. No studies reported changes in vital signs or biomarkers associated with dying. Barriers to the recognition of dying by healthcare professionals included a rapid and unpredictable terminal decline.

**Conclusions:**

Dying in motor neurone disease is associated with patterns of symptoms and signs, however evidence is limited compared with other terminal conditions and requires further exploration. The characteristic sudden and unpredictable terminal decline is a key barrier to recognition of dying by healthcare professionals. Optimising advance care planning is one approach to navigate these complex, unpredictable clinical situations.

**What is already known about the topic**

- Timely recognition of the dying phase enables optimal palliative care at the very end of life in patients with motor neurone disease.
- Healthcare professionals find it difficult to recognise dying and are often inaccurate in their predictions.
- Specific patterns of symptoms, signs and biomarkers are associated with approaching death in other conditions.

**What this paper adds**

- Dyspnoea, anxiety and pain were the most common symptoms at the end of life in motor neurone disease.
- Specific patterns of clinical features were identified as predictors that the dying phase was approaching.
- A sudden and unpredictable terminal decline was characteristic in motor neurone disease and was one of several barriers to timely recognition of dying by healthcare professionals.

**Implications for practice, theory or policy**

- Further research is needed to solidify knowledge on symptoms and signs at the end of life in motor neurone disease.
- Specific, measurable clinical signs and biomarkers associated with dying in motor neurone disease should be investigated, as has been done in other terminal conditions.
- Advance care planning should be optimised to plan for crisis situations and address the common clinical scenario of a sudden and unpredictable terminal decline.

## Introduction

The term motor neurone disease encompasses several conditions causing progressive degeneration of the body’s motor neurones, leading to muscle wasting and weakness.^1^ Disease progression typically leads to death within 3–5 years.^1,2^ Incidence of motor neurone disease has geographical heterogeneity, with over half of the prevalence and deaths occurring in high-income North America, Australasia and Western Europe.^2^ Despite a low incidence and prevalence, it is an illness with severe symptom burden and high fatality rate.^2^ In addition, the burden of motor neurone disease is increasing secondary to population aging.^2^ The complex physical and psycho-social impact of motor neurone disease, coupled with the typically poor prognosis, means that palliative care is an important component of managing this progressive, incurable illness.^3–5^

Recognising that the dying phase of an illness may be approaching is a key aspect of delivering the best care in any terminal condition.^6,7^ Accurate recognition can guide clinician decision-making about treatment (including treatment withdrawal and appropriate level of intervention escalation), inform communication with patient and family and facilitate individual priorities including preferred places of care and death.^8^ The importance of timely, accurate recognition of dying has been highlighted in several national and international guidelines.^7,9–11^ Despite this, we know that healthcare professionals find it difficult to recognise dying and are often inaccurate in their predictions.^12,13^ Emerging evidence suggests that specific patterns of clinical and biochemical features may predict the dying phase in certain patient populations.^14–17^ In patients with cancer, for example, the dying phase is associated with changes in respiratory and renal function, as well as potential biomarkers such as urinary volatile organic compounds.^15,17,18^ Clinician recognition of dying may also be influenced by their role and seniority, knowledge of the individual patient and collaborative discussion with other members of the multidisciplinary team.^16^

In the management of motor neurone disease, timely identification of dying enables important decisions to be made. These include the continuation or withdrawal of treatments, such as non-invasive ventilation and gastrostomy feeding, and prescription of anticipatory medications.^3^ A recent systematic review of forty studies examined palliative care needs in motor neurone disease and found that carers are sometimes taken by surprise by the dying phase, leading to deaths occurring unexpectedly and associated with distress.^5^ Carers sometimes reported features which, with hindsight, may have indicated that the dying phase was approaching.^5^ Further understanding about these features, and the perspectives of other stakeholders, such as healthcare professionals, would help us more accurately recognise that patients with motor neurone disease are entering the dying phase of their illness, and thus optimise palliative care provision and reduce distress.

The aim of this scoping review was to examine and map out what is known about dying in patients with motor neurone disease, and the recognition of dying by healthcare professionals. This includes recognisable physiological changes, patterns of deterioration, events which may predict dying, and any clinical and biochemical changes which may occur as end of life approaches. A scoping review enables us to understand the breadth of existing knowledge in this area and identify gaps in current evidence. This will help inform practice and guide future priorities for research within this area.^19,20^

## Methods

### Literature review question

The specific question to be addressed by the scoping review was:

What specific factors (clinical and biochemical features) enable healthcare professionals to recognise dying in patients with motor neurone disease?

An additional sub question was:

What are the barriers and facilitators to the recognition of dying in patients with motor neurone disease?

### Design

A scoping review methodology was chosen as an appropriate means to address our research questions, by mapping relevant key concepts, forms of evidence and gaps in the research, in an area which is so far undeveloped.^19,21^ The review was conducted according to the five stages described in the Arksey and O’Malley framework; identifying the research question, identifying relevant studies, study selection, charting the data, and collating, summarising and reporting the results.^21,22^ In line with more contemporary guidance our research question was broad, our scope clearly stated, and a sub-question was utilised to further examine the topic.^19,23,24^ The review has been reported according to the PRISMA extension for scoping reviews (PRISMA-Scr).^19,23,25^ It has been registered with OSF Registries and can be accessed at the following address: https://doi.org/10.17605/OSF.IO/J8FBE.

### Search strategy

A search strategy was developed in collaboration with an experienced medical librarian and adapted for each database, including key words and index terms (Supplemental File 1). It consisted of three main concepts generated from the research aim: the diagnosis of motor neurone disease and its subtypes; the dying process; and recognition or diagnosis. The term ‘death’ was not included in the search strategy, as its inclusion resulted in a high number of irrelevant publications, for example focussing on mortality data. Alternative terms were used to capture relevant papers, for example ‘last days of life’, whilst terms such as ‘palliative care’ ensured relevant articles were not excluded.

For the purposes of this review, ‘dying’ was considered as the last 2 weeks before death. This encompasses clinical terminology regarding the final ‘days’ of life,^9^ existing research into the recognition of dying,^15^ and is in keeping with the average 8.8 days duration of the terminal phase in patients with progressive neurological conditions.^26^

Databases searched were Ovid MEDLINE, PsycINFO, CINAHL and Scopus. These represent major healthcare databases, including those with a medical, nursing and psychological focus. A search of the grey literature was also undertaken; searches of Google, EThOS, Explore at the British Library, Open Grey and Grey Lit were completed using the terms ‘motor neurone disease’ AND ‘dying’. Websites of relevant organisations including the Motor Neurone Disease Association were also reviewed for information on this topic. Review articles were not included, but reference lists of relevant review articles and all included studies were examined to identify any additional relevant papers.

### Inclusion and exclusion criteria

Specific inclusion and exclusion criteria were used to inform study selection (Textbox 1).

**Textbox 1.** Inclusion and exclusion criteria.

Inclusion criteria • Empirical research (any design) • Focus on dying in motor neurone disease; physiological, clinical and biochemical changes, patterns of deterioration, events which may predict dying and its recognition by healthcare professionals • Relates to the last 2 weeks of life, or ‘dying’, ‘actively dying’, ‘dying phase’, ‘terminal phase’, or ‘end of life’ • Relates to motor neurone disease (amyotrophic lateral sclerosis, progressive bulbar palsy, progressive muscular atrophy, primary lateral sclerosis) • Focus on adults ⩾18 years old
Exclusion criteria • In language other than English • Focus on recognition of dying by family members and caregivers • Relates to spinal muscular atrophy • Editorials, commentary or opinion pieces, conference abstracts, case series, case reports, books.

An electronic literature search was conducted on 10th May 2023 with no date limits. Papers published at any time were included to maximise the comprehensiveness of the search. Titles and abstracts were initially screened by two independent reviewers (EA and MA). A full text review of any potentially eligible studies was then conducted by the same reviewers independently. Any disagreements at each stage were resolved through discussion with a third reviewer (PT).

### Data extraction

Data extraction was completed by EA using a proforma, designed by the research team and piloted before use (Supplemental File 2), and then verified by a second reviewer (CRM or PT). Data was mapped out in a descriptive manner according to the following: country, setting, population characteristics, aim/s, methods and findings. Findings were mapped to the key components of the research question: factors impacting the recognition of dying in motor neurone disease by healthcare professionals, clinical and biochemical features of dying in motor neurone disease; and barriers and facilitators to the recognition of dying in motor neurone disease by healthcare professionals.

### Collating and summarising the data

Findings were reviewed, and common themes identified by EA using constant comparative analysis. Following review and discussion with the research team, the following final themes were agreed;

- Clinical and biochemical features of dying in patients with motor neurone disease
  - Symptoms at the end of life
  - Signs of approaching death
  - Circumstances surrounding death
- Barriers and facilitators to the recognition of the dying phase in motor neurone disease by healthcare professionals

In keeping with the remit of the purpose of a scoping review, specific quality appraisal was not conducted.

## Results

### Search results

**Figure 1.**
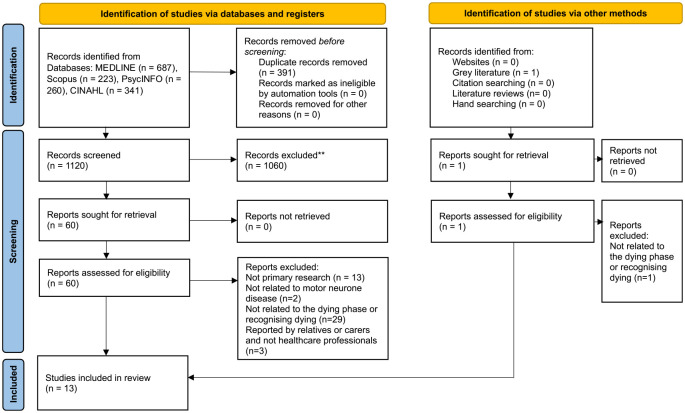
Flow diagram for the scoping review process.

From 1512 initial search results, 1120 were screened for eligibility, of which 13 were included in the scoping review. The screening process is outlined in Figure 1.

### Characteristics of included studies

The 13 included studies were conducted across 8 countries: the UK (*n* = 3),^27–29^ Sweden (*n* = 3),^30–32^ Japan (*n* = 2),^33,34^ Republic of Ireland (*n* = 1),^35^ Finland (*n* = 1),^36^ Norway (*n* = 1),^37^ Canada (*n* = 1)^38^ and one multicentre study involving Germany and the UK (*n* = 1).^39^ The majority of studies were quantitative in nature (*n* = 7)^28–32,35,36^ using retrospective reviews of medical records, registry data and other databases. A minority used qualitative methods only (*n* = 4),^27,34,37,38^ utilising interviews, focus groups or both and the remainder used mixed-methods (*n* = 2).^33,39^

Seven of the studies focussed on patient data alone (*n* = 7),^28–32,35,36^ two collected data from healthcare professionals alone (*n* = 2),^34,38^ and the remainder collected data from a combination of; patients and carers (*n* = 1),^39^ patients and healthcare professionals (*n* = 1),^33^ and carers (*n* = 1),^27^ and patients, carers and healthcare professionals (*n* = 1).^37^ In each paper, the data was presented separately, allowing the results relevant to the scoping review question to be extracted. As our specific focus was the recognition of dying by healthcare professionals, data from family members and carers was not included in our results.

Studies were based in a hospice (*n* = 2),^29,36^ outpatient clinics (*n* = 1),^28^ and a home care nursing agency (*n* = 1).^34^ Two utilised national databases alone (*n* = 2).^30,31^ The remainder recruited from a mixture of these settings (*n* = 6),^32,33,35,37–39^ and in one paper the setting was unclear (*n* = 1).^27^

All 13 studies reported clinical features of dying in motor neurone disease. No studies described biochemical features of dying in motor neurone disease. Five studies reported symptoms at the end of life (*n* = 5),^30–32,36,39^ and two described clinical signs which may predict the end of life is approaching (*n* = 2).^34,38^ Nine studies detailed wider circumstances surrounding death in motor neurone disease (*n* = 9).^27–30,32–35,39^ Whilst no studies described facilitators to the recognition of dying in motor neurone disease by healthcare professionals, four studies reported barriers (*n* = 4).^27,34,37,38^ Table 1 summarises the characteristics of the included studies.

**Table 1.** Characteristics of studies included in the scoping review.

First author	Title	Aim/s	Location/setting	Method/population	Data presented relevant to scoping review
Baxter et al.^27^	The Use of Non-Invasive Ventilation at End of Life in Patients with Motor Neurone Disease: A Qualitative Exploration of Family Carer and Health Professional Experiences	To describe carer and healthcare professional experiences of end of life care of motor neurone disease patients using NIV.	UK. Motor neurone disease clinic within single hospital.	Qualitative. Longitudinal semi-structured interviews. Family carers (*n* = 9) and healthcare professionals (*n* = 15) who were closely involved in the final months of life of patients with motor neurone disease who were using NIV during the final phase of their disease.	Barriers to recognising dying. Speed of terminal decline.
Chaudri et al.^28^	Patterns of Mortality in Patients with Motor Neurone Disease	To explore patterns of mortality in patients with motor neurone disease with an emphasis on place of death.	UK. Motor neurone disease clinic within single hospital.	Quantitative. Retrospective review of hospital and GP medical notes, and post-mortem reports. All patients (*n* = 179) at the clinic with probable or definite motor neurone disease who died between 1990 and 2000.	Cause of death. Place of death.
Daneau et al.^38^	‘Intensive Palliative Care’: A Qualitative Study of Issues Related to Nurses’ Care of People with Amyotrophic Lateral Sclerosis at End-of-Life.	To explore the experience of nurses when caring for people with ALS at end-of-life. To explore the characteristics of nurses’ care of people with ALS at end-of-life. To identify the needs of nurses in providing quality care to people with ALS at the end-of-life.	Canada. Home care, hospitals and palliative care homes in Quebec.	Qualitative. Individual semi-structured interviews. Nurses (*n* = 24) from hospitals, home care and palliative care homes who had provided care for at least one person with ALS at the end of life in the last 12 months.	Barriers to recognising dying. Signs at end of life.
Eljas Ahlberg and Axelsson^30^	End-of-Life Care in Amyotrophic Lateral Sclerosis: A Comparative Registry Study	To study whether the quality of end of life care in the last week of life for patients dying from ALS differed compared to patients dying from cancer. To learn more about the place of death for patients with ALS in Sweden.	Sweden. Used data from the Swedish Register for Palliative Care and the Swedish Cause of Death Register.	Quantitative. Retrospective comparative registry study. Patients (*n* = 825) who had an expected death, with ALS reported as the main cause of death in the SRPC database, and/or in the Swedish Cause-of-Death Register 2012–2016. Patients (*n* = 3300) with cancer reported as the main cause of death in the SRPC during the same period were chosen as controls.	Symptoms at end of life.
Lerum et al.^37^	Unstable Terminality: Negotiating the Meaning of Chronicity and Terminality in Motor Neurone Disease	To explore the meaning of chronicity and terminality in MND.	Norway. Three sites, each consisting of a hospital and corresponding primary care.	Qualitative. Narrative and thematic interviews. Patients with motor neurone disease (*n* = 5) and their informal carers (*n* = 25). Healthcare professionals from primary (*n* = 18) and secondary (*n* = 17) care with experience looking after patients with motor neurone disease.	Barriers to recognising dying. Speed of terminal decline.
Neudert et al.^39^	The Course of the Terminal Phase in Amyotrophic Lateral Sclerosis	Not explicitly stated; implies aim is to understand the natural course of death in patient with ALS.	UK and Germany. Motor neurone disease clinic in single German hospital, and single UK hospice.	Mixed methods. Retrospective analysis of patient records from German and UK hospitals. Structured interviews with relatives of patients recruited from the German hospital. Patients (*n* = 121) with motor neurone disease who died January 1995–March 1999 from motor neurone disease clinic list at German hospital, and caregivers who were present at the moment of death of for most of the 24 h before death. Patients (*n* = 50) with ALS who died between 1991 and 1999 who had been followed by the UK hospice.	Cause of death. Speed of terminal decline. Symptoms at end of life.
O’Brien et al.^29^	Motor Neurone Disease: A Hospice Perspective	To describe and evaluate management of patients with motor neurone disease from a hospice perspective.	UK. Single hospice.	Quantitative. Retrospective review of medical and nursing notes. Patients (*n* = 124) with motor neurone disease cared for by the hospice January 1980–November 1990.	Cause of death. Speed of terminal decline.
Ozanne et al.^31^	Symptom Relief During Last Week of Life in Neurological Disease	To investigate symptom prevalence, symptom relief and palliative care indicators in the last week of life.	Sweden. Used data from the Swedish Register for Palliative Care and the Swedish Cause of Death Register.	Quantitative. Retrospective comparative registry study. Patients aged 18 or over whose cause of death was ALS (*n* = 419), CNS tumour (*n* = 799) or other neurological disease (*n* = 1407) between January 2011 and December 2012.	Symptoms at end of life.
Ryan et al.^35^	A Retrospective Review of Specialist Palliative Care Involvement in Motor Neurone Disease	To describe the demographic and clinical characteristics of motor neurone disease patients referred to hospice services for palliative care assessment. To collect peri-mortem data regarding these patients. To understand how these patients used the hospice services.	Republic of Ireland. Hospice services serving two areas of Dublin; comprising a home-care service, an inpatient service, a day hospice service and an outpatient service.	Quantitative. Retrospective review of clinical notes. All patients (*n* = 72) with a diagnosis of motor neurone disease that had their first palliative care assessment between 1/1/99 and 31/12/08.	Place of death.
Sennfält et al.^32^	Dying from ALS in Sweden: Clinical Status, Setting, and Symptoms	To provide a comprehensive account of death in patients with ALS, including preceding clinical status, place of death and symptoms.	Sweden. Single hospital in Stockholm. Also used data from Swedish Motor Neurone Disease Quality Registry and Swedish Register of Palliative Care.	Quantitative. Review of patient medical records, the Swedish Motor Neuron Disease Quality Registry and the Swedish Quality Registry of Palliative Care. Two cohorts. Main cohort of patients (*n* = 93) diagnosed with ALS at study hospital 2016 onwards, deceased 2018–2020 and registered with the Swedish Motor Neurone Disease Quality Registry and the Swedish Quality Registry of Palliative Care. Secondary cohort of patients (*n* = 2224) diagnosed with ALS in any region of Sweden, that died 2011–2020 and registered with the Swedish Quality Registry of Palliative Care.	Place of death. Symptoms at end of life.
Tiirola et al.^36^	End-of-Life Care of Patients with Amyotrophic Lateral Sclerosis and Other Nonmalignant Diseases	To explore the diagnoses, symptoms and treatment of patients dying in a hospice due to nonmalignant diseases, particularly ALS.	Finland. Single hospice.	Quantitative. Retrospective analysis of patient records. Patients (*n* = 67) with non-malignant diseases who died in the hospice 2004–2013, of which a proportion had ALS (*n* = 32).	Symptoms at end of life.
Ushikubo et al.^33^	Illness Course and Circumstances of Death among Individuals with Rapidly Progressive Amyotrophic Lateral Sclerosis	To understand the illness course and circumstances of death in individuals with ALS who died within 1 year of starting home care.	Japan. Single home care nursing agency, and the neurology ward of a university hospital.	Mixed methods. Retrospective analysis of hospital patient records. Minimally structured interviews with nursing staff. Patients (*n* = 6) who were admitted to the neurology ward and diagnosed with ALS April 2009–March 2011, started homecare nursing and died within 1 year of leaving hospital. Nurses from the homecare nursing agencies providing care for these patients (*n* = 5). Nurses from the hospital discharge liaison team (*n* = 3).	Cause of terminal decline.
Ushikubo^34^	Circumstances and Signs of Approaching Death in Patients with Amyotrophic Lateral Sclerosis Undergoing Noninvasive Ventilation in Home Care Settings	To ascertain the circumstances and symptoms of patients with ALS using NPPV approaching death, to understand how to provide palliative care to these patients.	Japan. Six home care nursing agencies within one region of Japan.	Qualitative. Individual and group semi-structured interviews. Home visiting nurses (*n* = 6) who were the head or subhead of nursing agencies with experience of caring for patients with ALS.	Barriers to recognising dying. Cause of terminal decline. Signs at end of life.

Study results are displayed in Tables 2–5. Studies which have findings relating to more than one area are reported separately in each appropriate table.

**Table 2.** Symptoms present at the end of life in motor neurone disease.

Symptoms present at the end of life	Prevalence in cited study
Dyspnoea	58.3%,^31^ 57.5%,^30^ 48%,^36^ 30%,^39^ present but prevalence not specified^32^
Anxiety	64.6%,^30^ 62.7%,^31^ 13%,^36^ present but prevalence not specified^32^
Pain	52.7%,^31^ 49.7%,^30^ 42%,^36^ present but prevalence not specified^32^
Difficulty swallowing	87%^36^
Retained respiratory secretions	58.1%^31^
Fatigue	42%^36^
Coughing	20%^39^
Confusion	14.6%,^31^ present but prevalence unspecified^32^
Nausea	12.3%,^31^ 5%^36^
Diffuse pain	2%^39^
Restlessness and anxiety	6%^39^
Constipation	3%^36^
Choking on saliva or mucus	0%^39^
Excessive bronchial secretions	Present but prevalence not specified^32^

**Table 3.** Signs of approaching death in patients with motor neurone disease.

Deterioration in respiratory function;^38^
• Repeated respiratory infections at increasingly shorter intervals^34^
• Respiratory distress when patient moved^34^
Development of new symptoms
• Difficulty digesting enteral nutrition, causing increased gastric residual volume or reflux^38^
• Weight loss^34^
• New oral complications^34^
• New skin breakdown^34^
Frequent adjustments of medication to manage symptoms without achieving stability^38^

**Table 4.** Circumstances surrounding death in motor neurone disease.

Cause of terminal decline/death
• Commonly acute or acute on chronic respiratory failure^29^
• 43% died from pneumonia, 31% from respiratory failure, 14% from general deterioration, 7% from cardiac causes, 2% from saddle embolus and 2% from a perforated duodenal ulcer ^28^
• 96% patients died of respiratory failure, and 4% died of heart failure^39^
• Death occasionally occurred from clinical incident, for example NIV machine failure, or dislocation of face mask^34^
• Death was sometimes influenced by carer ability, for example difficulties using machinery^33^
• No evidence for choking as a cause of death^27,29,39^
Speed of terminal decline
• 40% deteriorated suddenly and died within 12 h; 18% within 24 h; 24% within 3 days and 17% within 7 days^29^
• Variable, evenly spread between 0.5 h and 168 h^39^
• Health care professionals described the end of life phase as unexpectedly fast^27^
• Rapid clinical change described as ‘galloping’^37^
Place of death
• 45% died at home, in a hospice or in a nursing home, and 36% died in hospital^28^
• In 9% of deaths which occurred in hospital, the admission was deemed avoidable^28^
• 36% died at home, 36% died in the inpatient unit, 15% died in hospital and 13% died in a nursing home^35^
• 93.4% anticipated/prolonged deaths occurred in a palliative care unit, at home, or in an assisted living facility. 44.8% of precipitous deaths occurred in a hospital ward^32^

**Table 5.** Barriers to the recognition of dying in motor neurone disease by healthcare professionals.

Barrier	Illustrative quotes
Rapid terminal decline	*‘It felt like a more sudden and dramatic end than I had imagined. I had imagined being able to guide the family through it in a bit more of a controlled way than when it eventually happened’.* ^27^ *‘He/she answered that he/she was not in pain at all when I asked if he/she felt pain or not, but he/she worsened and died’.* ^34^ *‘Many of the participants, both carers and professionals, underscored that MND develops fast. ‘Galloping’ was a metaphor participants used several times’* ^37^
Variable clinical pattern of deterioration between patients	*‘You know, everyone has a different trajectory, no matter what the disease is, but my impression is that the ALS trajectory is different for everyone too . . . and it’s like impossible for me to say, “Well there’s this much time left or there’s that much time left. . .”’* ^38^
Dying phase presents differently compared to other diseases	*‘You know, I mean, there are symptoms that my oncology patients have that make me think they’re not going to last long. My ALS patients are the same but they’re not at the end-of-life’.* ^38^
Repeated episodes of recovery	*‘He/she was coughing up a lot of phlegm or had repeated pneumonia, but every time he/she recovered from it following administration of antibiotics’.* ^34^ *‘I often thought he/she will perhaps die this time but he/she recovered from that serious situation’* ^34^

ALS: amyotrophic lateral sclerosis; MND: motor neurone disease.

Terminology and abbreviations are as used in the original paper.

Terminology and abbreviations are as used in the original paper. ALS: amyotrophic lateral sclerosis; CNS: central nervous system; GP: general practitioner; MND: motor neurone disease; NIV: non-invasive ventilation; NPPV: non-invasive positive pressure ventilation; UK: United Kingdom

## Clinical and biochemical features of dying in motor neurone disease

### Symptoms at the end of life

Five studies recorded symptoms present in the dying phase (Table 2), either in the last week of life,^30–32^ or in the last 24 h of life.^36,39^ No studies reported on biochemical features. High levels of dyspnoea, anxiety and pain were reported in five studies (range from 30% to 64.6%). Difficulty swallowing, whilst only reported in one study, was experienced by the highest proportion (87%) of patients compared with any other symptom. It was noteworthy that the most commonly reported medical treatments used within the terminal phase were opioids (range 82%–90.6%) and benzodiazepines (range 60%–71.9%).^29,35,36,39^ Administration of medication using a syringe driver was reported in over 75% of patients.^35^ Laxatives, antibiotics and riluzole were also frequently used on the last day of life (75%, 29%–31% and 45% respectively).^35,36^

### Signs of approaching death

Two papers reported on patterns of clinical signs which might suggest the approach of the dying phase (Table 3).^34,38^ Both studies reported a deterioration in respiratory function as a key sign that death was approaching. Repeated respiratory infections at shortening intervals and respiratory distress on movement were specifically noted.^34^ Approaching death was also noted to be associated with the development of certain new symptoms; increased reflux, weight loss, oral complications and skin breakdown.^34,38^ Finally, a deterioration in symptom control despite optimal medical management was a further sign that the end of life was approaching.^38^

### Circumstances surrounding death

Nine papers described circumstances surrounding death in patients with motor neurone disease (Table 4).^27–29,32–35,37,39^ Five studies reported patients’ cause of death or terminal decline.^28,29,33,34,39^ ‘Respiratory failure’ was accountable for death in 31%–96% patients (with frequency not specified in a further study).^28,29,39^ In addition, pneumonia, and saddle embolus, which would also be expected to cause respiratory failure, were reported as cause of death in 43% and 2% respectively.^28^ General deterioration was reported as a cause of death in 14% cases in one study, with cardiac causes, and specifically heart failure, less frequently noted. Clinical incidents, such as face mask dislocation, were occasionally noted as the cause of terminal decline.^33,34^ Patients may fear choking at the end of life, however three studies demonstrated no evidence for choking as a cause of death in motor neurone disease.^27,29,39^ One study included a postmortem series of a subset of 19 patients, at least one of which was prompted by a concern about choking.^29^ In none of these cases was foreign matter found in the airway, and no evidence of choking was recorded in the wider cohort.^29^

Four studies commented on the rapid nature of the terminal decline, often sudden and unexpected, and the unique nature of this decline compared to other conditions.^27,29,37,39^ One study found the majority of patients died within 0.5 h and 7 days of an acute deterioration, with no clear peak between these times.^39^ A second study reported that almost half of patient deteriorated suddenly and died within 12 h.^29^ When describing the dying process in patients with motor neurone disease, healthcare professionals used terms such as ‘unexpectedly fast’ and ‘galloping’.^27,37^

Patients with motor neurone disease were most likely to die at home or in a palliative care unit. One study identified that anticipated deaths were less likely to occur in hospital compared with patients who deteriorated more suddenly.^32^ In a minority of cases where patients died in hospital, the hospital admission was felt to be avoidable.^28^

## Barriers to the recognition of the dying phase in motor neurone disease by healthcare professionals

Four qualitative studies commented on specific barriers to recognising dying in patients with motor neurone disease by healthcare professionals.^27,34,37,38^ Their findings are described narratively below with illustrative quotes (Table 5). No studies reported facilitators to the recognition of dying.

Building on the previous recognition that clinical decline in motor neurone disease can be rapid and unpredictable, three studies explicitly cited this as a barrier to timely recognition of the end of life.^27,34,37^ Perhaps as a reflection of this clinical picture, patients may report feeling well and maintain a high functional level until just before death.^34^ These patterns led to difficulties judging patient prognosis and making decisions about appropriate treatment and escalation.

Motor neurone disease may present with a variable clinical picture both between different forms of motor neurone disease and between individuals with the same form.^38^The dying phase is also perceived by healthcare professionals as different to other terminal diseases, preventing accurate recognition.^27,38^ Severe cachexia, dysphagia and use of non-invasive ventilation, for example, might indicate imminent death in patients with cancer, but could be present for several weeks or longer in patients with motor neurone disease.^38^

Repeated episodes of acute illness and deterioration, followed by recovery, also made it difficult to know when a deterioration was likely to be a terminal event.^34^ This potentially could compound wider difficulties around the time of death which include poor collaboration between family physician and hospital physician, difficulty in management of psychological distress, deterioration of family relationships, poor understanding of the disease by patient and family, and difficulties in arrangements for home care support.^33^

## Discussion

### Main findings

This review highlights a number of clinical features associated with the dying phase in motor neurone disease. The symptoms most commonly reported in these patients are dyspnoea, anxiety and pain. Signs indicating the onset of the dying phase are deterioration in respiratory function (increased frequency of respiratory infections, increased respiratory secretions), failure of enteral feeding, the development of new symptoms (oral symptoms and skin breakdown) and poorly controlled symptoms despite regular adjustments of medications. Patients with motor neurone disease most frequently died at home or in a palliative care inpatient unit, with respiratory failure the most common cause of death.

A rapid, unpredictable decline is characteristic of the dying phase. This is a key barrier to the timely recognition of dying by healthcare professionals in this patient cohort. Additional barriers include variation in clinical trajectory between individual patients, and a differing clinical picture at the end of life compared with other life-limiting conditions.

### What this study adds and implications for practice

We know that there can be a failure to accurately recognise when the end of life is approaching in motor neurone disease, causing distress to patients and families.^5^ This scoping review is the first to map out what is known about dying with motor neurone disease, and its recognition by healthcare professionals.

Dyspnoea and pain were amongst the most common symptoms present in patients with motor neurone disease at the end of life. This is consistent with evidence that certain physical symptoms, including dyspnoea, pain, loss of appetite and increased dependency, are associated with the dying phase in both malignant and non-malignant conditions.^40–44^ Similarly, anxiety was common in patients dying with motor neurone disease, in keeping with psychosocial characteristics present in the dying phase of other illnesses such as difficulty coping, and struggling to find meaning and purpose in life.^40,45–47^

Our review also identified patterns of physical signs associated with approaching death in motor neurone disease. These are described in two qualitative studies comprising a total of thirty healthcare professionals.^34,38^ This is notably limited in scale and methodology when compared to other patient groups. In cancer care, for example, observational studies of hundreds of participants have demonstrated that specific physical signs predict impending death.^48–53^

We sought to identify studies which reported biochemical features associated with approaching death in motor neurone disease. Whilst certain biochemical features, such as biomarkers, are useful in the diagnosis and early prognostication of motor neurone disease, it is noteworthy that we found no studies reporting on such markers at the end of life in motor neurone disease.^54^ In contrast, a systematic review of thirty studies across fourteen countries demonstrated Grade A evidence for seven biomarkers as predictors of survival in advanced cancer.^17^ It also demonstrated more limited evidence for the association of several markers (white cell count, platelet count, CRP, urea, urate, alanine transaminase, lactate dehydrogenase, sodium and plasma interleukin-6) with the last 2 weeks of life.^17^ We suggest that exploring these principles in motor neurone disease would be a valuable focus of further research.

Our review identified the often rapid and unpredictable nature of the decline in motor neurone disease at the very end of life, which prevented healthcare staff feeling confident in recognising the onset of the dying phase.^27,34,37^ This pattern differs from the more prolonged ‘dwindling’ deterioration classically associated with frailty, and the period of evident decline typically seen in cancer.^55,56^ Whilst the trajectory seen in cancer is sometimes described as a sudden decline, our review suggested that the deterioration at the end of life in motor neurone disease is over a shorter time period, and that signs of dying in cancer are different to those in motor neurone disease.^45,55^ Some similarities may be drawn between the deterioration at the end of life in patients with motor neurone disease and those with organ failure, who typically display repeated episodes of crisis which may or may not be recoverable.^55^ Establishing robust advance care planning, thus preparing patients and family caregivers for crisis situations before they arise, is one approach to counter unexpected deterioration.^55–57^ We know that patients diagnosed with motor neurone disease think about topics such as end of life treatment, advance directives and goals of care within 1 month of diagnosis.^58^ This suggests the majority of patients may be comfortable discussing these topics with healthcare professionals and planning ahead.^58^ Introducing the concept of advance care planning at the point of diagnosis is acceptable to patients.^59^ It is therefore the responsibility of healthcare professionals to offer these discussions, and barriers to this, including lack of confidence, skill and time, must be addressed.^60^

### Strengths and limitations of the study

This scoping review was conducted according to an established, systematic method. The search strategy was designed in discussion with a senior librarian with expertise in literature searching. We conducted grey literature searching and screening of reference lists to minimise the chance of overlooking relevant articles. Data extraction was verified by a second reviewer to ensure consistency and reduce error. Studies were from a diverse range of countries and have relevance to palliative care internationally.

We have deliberately focused on the last 2 weeks of life, in order to capture events in the immediate time period preceding death. Nevertheless, we recognise that some studies of referenced longer time periods, for example 1 month, and hence certain papers that reflect changes leading up to death at longer time scales have been excluded. We limited our search to papers published in the English language.

## Conclusion

This scoping review has demonstrated that dying in motor neurone disease is associated with patterns of symptoms and signs, which may help healthcare professionals to recognise when the end of life is approaching. Evidence for these, however, is limited compared with other terminal conditions, and further clarity is needed with particular focus on specific measurable features, such as vital signs and biomarkers. The sudden and unpredictable nature of the terminal decline in motor neurone disease is a key barrier to its recognition by healthcare professionals, and the optimal delivery of care during the dying phase. A focus on optimising advance care planning is one approach to navigate these complex and unpredictable clinical situations.

## Supplemental Material

sj-docx-1-pmj-10.1177_02692163241263231 – Supplemental material for Recognising dying in motor neurone disease: A scoping review Supplemental material, sj-docx-1-pmj-10.1177_02692163241263231 for Recognising dying in motor neurone disease: A scoping review by Elizabeth Abbey, Maimoona Ali, Matthew Cooper, Paul Taylor and Catriona R Mayland in Palliative Medicine

sj-docx-2-pmj-10.1177_02692163241263231 – Supplemental material for Recognising dying in motor neurone disease: A scoping review Supplemental material, sj-docx-2-pmj-10.1177_02692163241263231 for Recognising dying in motor neurone disease: A scoping review by Elizabeth Abbey, Maimoona Ali, Matthew Cooper, Paul Taylor and Catriona R Mayland in Palliative Medicine
