# Supplementary material for: Recognising dying in motor neurone disease: A scoping review
Source: Palliat Med. 2024 Jul 28;38(9):923–34. doi: 10.1177/02692163241263231 (PMC11481408; doi:10.1177/02692163241263231)
Supplement: sj-docx-1-pmj-10.1177_02692163241263231 – Supplemental material for Recognising dying in motor neurone disease: A scoping review [file sj-docx-1-pmj-10.1177_02692163241263231.docx]

**Supplementary file 1.** Example search strategy from MEDLINE by Ovid.

| **Set** | **Search Statement** |
| --- | --- |
| 1 | \|  \| ("motor neurone disease" or "motor neuron disease" or "amyotrophic lateral sclerosis" or "progressive bulbar palsy" or "pseudobulbar palsy" or "progressive muscular atrophy" or "primary lateral sclerosis" or "Lou Gehrig’s disease" or "MND" or "ALS").mp. [mp=title, abstract, original title, name of substance word, subject heading word, floating sub-heading word, keyword heading word, organism supplementary concept word, protocol supplementary concept word, rare disease supplementary concept word, unique identifier, synonyms] \| \| --- \| --- \| |
| 2 | exp Motor Neuron Disease/ |
| 3 | ("dying" or "terminal phase" or "terminal care" or "terminally ill" or "end of life" or "palliative care" or "palliative medicine" or "hospice and palliative nursing" or "hospice care" or "last days of life" or "last hours of life" or "last week of life" or "final days of life" or "final hours of life" or "final week of life").mp. [mp=title, abstract, original title, name of substance word, subject heading word, floating sub-heading word, keyword heading word, organism supplementary concept word, protocol supplementary concept word, rare disease supplementary concept word, unique identifier, synonyms] |
| 4 | exp Terminal Care/ |
| 5 | exp Palliative Care/ |
| 6 | exp Hospice Care/ |
| 7 | \|  \| ("recognition" or "recognizing" or "recognize" or "recognising" or "recognise" or "diagnosing" or "diagnosis" or "diagnose" or "assess" or "tool" or "instrument" or "criteria" or "pathway" or "signs" or "symptoms" or "observation" or "perception" or "criteria" or "identify" or "identification" or "identifying" or "assess" or "assessment" or "assessing" or "prognosis prognostic" or "predictor" or "predict" or "predicting" or "biochemical marker" or "clinical marker" or "laboratory marker" or "biomarker" or "biological marker").mp. [mp=title, abstract, original title, name of substance word, subject heading word, floating sub-heading word, keyword heading word, organism supplementary concept word, protocol supplementary concept word, rare disease supplementary concept word, unique identifier, synonyms] \| \| --- \| --- \| |
| 8 | exp Diagnosis/ |
| 9 | 1 or 2 |
| 10 | 3 or 4 or 5 or 6 |
| 11 | 7 or 8 |
| 12 | 9 and 10 and 11 |
| 13 | \|  \| limit 12 to (english language) \| \| --- \| --- \| |
