# Supplementary material for: Recognising dying in motor neurone disease: A scoping review
Source: Palliat Med. 2024 Jul 28;38(9):923–34. doi: 10.1177/02692163241263231 (PMC11481408; doi:10.1177/02692163241263231)
Supplement: sj-docx-2-pmj-10.1177_02692163241263231 – Supplemental material for Recognising dying in motor neurone disease: A scoping review [file sj-docx-2-pmj-10.1177_02692163241263231.docx]

**Supplementary file 2. Data extraction tool**

|  | **Results** |
| --- | --- |
| **Title** |  |
| **Author** |  |
| **Year of publication** |  |
| **Country of origin** |  |
| **Citation** |  |
| **Setting** |  |
| **Aims / purpose** |  |
| **Study population and size** |  |
| **Type of study / methods** |  |
| **Limitations** |  |
| **Key findings that relate to scoping review question(s)**  “How do HCPs recognise dying in patients with MND?” |  |
| **Additional sub-question** |  |
| What are the clinical and biochemical markers of dying in MND? |  |
| What are the barriers and facilitators to the recognition of dying in patients with MND? |  |

**Additional comments:**

**Reference list**
